# A new scoring system to predict fatal accidents in General Aviation and to facilitate emergency control centre response

**DOI:** 10.1038/s41598-024-77994-3

**Published:** 2024-11-14

**Authors:** Jochen Hinkelbein, Catherina Hippler, Felix Liebold, Jan Schmitz, Markus Rothschild, Volker Schick

**Affiliations:** 1 Department of Anaesthesiology, Intensive Care Medicine, Emergency Medicine and Pain Medicine, Johannes Wesling University Hospital Minden, Ruhr-University Bochum, Hans-Nolte-Straße 1, 32429 Minden, Germany; 2 German Association of Aerospace Medicine (DGLRM), Munich, Germany; 3 European Society of Aerospace Medicine (ESAM), Cologne, Germany; 4 Department of Anaesthesiology Und Intensive Care Medicine, Faculty of Medicine and University Hospital Cologne, University of Cologne, Cologne, Germany; 5 Department of Anaesthesiology and Intensive Care Medicine, University Hospital and Faculty of Medicine Leipzig, Leipzig, Germany; 6 Department of Sleep and Human Factors Research, German Aerospace Center, Cologne, Germany; 7 Institute of Legal Medicine, Faculty of Medicine and University Hospital Cologne, University of Cologne, Cologne, Germany

**Keywords:** Flight accidents, General Aviation, Scoring system, Health care, Risk factors

## Abstract

Numerous accidents occur with General Aviation aircraft every year. To date, pre-emptive prediction of survival or death is impossible. The current study aims to identify significant factors elementary to predict survival after General Aviation (GA) aircraft accidents. The Implementation of a scoring system, including these factors, may facilitate emergency control centre response. Data of flight accidents over a 20-year period (extracted from the German Federal Bureau of Aircraft Accident Investigation [BFU]) was analysed for fixed-wing motorized small aircrafts below 5,700 kg MTOW. Factors of interest were analysed using Chi^2^- and Mann–Whitney-U-Tests. Logistic regression was used to establish a score to calculate the probability of a fatal outcome after an aircraft accident. The BFU lists 1,595 GA aircraft accidents between 2000 and 2019. The factors “third quarter of the year” (p = 0.04), “last quarter of the year” (p = 0.002), “fire” (p < 0.0001), “distance from airport > 10 km” (p < 0.0001), “landing” (p < 0.0001) and “cruise” (p < 0.0001), significantly correlated positively or negatively with a fatal outcome. “Take-off”, “approach”, “month”, “day of the week”, “persons on board above three”, “night-time” and “icing conditions” showed no significant correlation. Using logistic regression “third quarter of the year” and “cruise” were excluded when using the B-STEP method. Including the four significant parameters, the score showed a strong effect with f^2^ = 0.709. The analysis of GA aircraft accidents in Germany enabled the identification of relevant factors and establishment of a new scoring system for survival prediction after small aircrafts accidents below 5,700 kg MTOW. The implementation of the scoring system in emergency control centres in the context of digital development and artificial intelligence can improve emergency response planning and distribution.

## Introduction

General aviation (GA) comprises all civilian air transportation other than commercial passenger transportation or charter operations. In terms of number of aircrafts and aircraft movements, GA is the largest sector of aviation. One quarter of GA aircraft is flown for recreational purposes by private pilots, the other three quarters include flight instruction, business travel, and emergency medical flights^1^.

Despite a decline in recent years, the rate of GA accidents is substantially higher as compared with airline operations^2^. The number of accidents involving GA, documented by the National Transportation Safety Board (NTSB), decreased from 1,728 in 2001 to 1,085 accidents in 2020. However, the percentage of fatal outcomes remained unchanged, around 18%^3^. The International Civil Aviation Organization defines aircrafts by their maximum take-off weight (MTOW) as small aircraft < 5,700 kg and large aircrafts ≥ 5,700 kg^4^.

Analysis of aircraft accidents and their influencing factors is an elementary part of aviation- and emergency medicine. In contrast to the large aircrafts, the use of a flight data recorder ("Flight Data Monitoring", FDM) is not mandatory for small aircraft. As a result, accident analysis is based on observable factors such as weather conditions, witness statements, the investigator’s assessment, fires, or aircraft wreckage^5^. Through this, predictions of injury and survival probability can be made to improve flight safety in the future^6^.

In areas of emergency and critical care medicine, scoring systems are a crucial tool for estimating mortality risks^7^. Injury and survival probabilities have been successfully predicted within the clinical setting for over 50 years. Regarding aviation medicine, Li et al. (2008) were able to identify three main risk factors leading to a fatal outcome after an aircraft crash. Based on these three factors the FIA score was developed 20 years ago^8^. It reports that Fire (F), instrument flight rules/weather (I) and the distance away from an airport (A) are major contributing factors for General Aviation accidents.

The goal of the current study is to identify significant factors that correlate with serious or fatal outcomes in the context of small aircraft accidents and the development of an outcome scoring system. This score is developed specifically for use in rescue coordination centres and includes query-able factors.

## Methods

### Procedure and data collection

Data were obtained from a retrospective search of the online database of the German Federal Bureau of Aircraft Accident Investigation (Bundesstelle für Flugunfalluntersuchung – BFU) for annual accident statistics and detailed accident reports. All accident reports involving motorized small aircrafts between 2000 and 2021 were included (last data collection on 06.02.2021).

This study focussed on the aircraft classes < 2,000 kg and 2,000 kg – 5,700 kg MTOW. In total, 338 accident reports were found for the mentioned period, of which 275 could be assigned to the aircraft class < 2,000 kg and 63 to the class 2,000 kg – 5,700 kg MTOW. Referring to the definition of accidents and incidents according to the Convention on International Civil Aviation^9^, a detailed analysis of all "accidents" was performed, while listed "incidents" were excluded.

Furthermore, accidents which were investigated by non-German authorities were excluded. Events involving two aircrafts were considered as two separate accidents. After excluding the above criteria, 285 (MTOW < 2,000 kg = 238, MTOW 2.000 kg – 5,700 kg = 47) accident reports could be included in this study.

Since the current study relies on recently published data in the public domain, no ethics commission approval or institutional review board was required.

### Statistical analysis

Specific parameters were recorded and further analysed to generate descriptive statistics as well as inferential statistical procedures (Table 1). Statistical analysis was performed using Chi^2^ -Tests and Mann–Whitney-U-Test to determine if dichotomized parameters correlated with the outcome fatal injury. P-values less than 0.05 were considered statistically significant.

**Table 1:** Examined categories and subcategories with highlighted core parameters.

Categories	Subcategories
Data	Date [DD MM JJJJ]
	Year [E.g. 2000]
	Month [January – December]
	Season [Spring, Summer, Autumn, Winter]
	Quarter [first-, second-, third- and last quarter of the year]
	Weekday [Monday—Sunday]
	Weekend [Friday-Sunday]
Type of emergency	Engine failure [yes, no]
	Fire [yes, no]
Aircraft parameters	Weight Category [≤ 2000 kg MTOW, 2000 – 5700 kg MTOW]
	Multi-engine [yes, no]
	Single-engine [yes, no]
	Multi_Single_Engine [Multi-engine, Single-engine]
	Number of seats [Quantity]
	Seats > 4 [yes, no]
	Number of persons on bord [Number]
	Number of persons on bord ≤ 3 [yes, no]
	Number of persons on bord > 3 [yes, no]
	Gear [retractable, fixed]
	Aircraft Type [name]
	Diamond Aircraft Industries [yes, no]
	Cessna Aircraft Company Inc. [yes, no]
	Piper Aircraft, Inc. [yes, no]
	Beechcraft Corporation [yes, no]
	Aircraft type other [yes, no]
	Weight of aircraft at time of accident [kg]
	Maximum take-off weight (MTOW) [kg]
Location and flight phase	Distance away from runway [< 10 km, > 10 km]
	Flight phase [Take-off, cruise, approach, landing]
Pilot factors	Cockpit Crew [Quantity]
	Pilot experience—total [hours]
	Pilot experience – on this type of aircraft [hours]
	Pilot licence [PPL, CPL, SPL, LAPL, ATPL]
	PPL [yes, no]
	CPL [yes, no]
	SPL, LAPL [yes, no]
	ATPL [yes, no]
	Commercial or private pilote licence [Commercial pilot licence, private pilot licence]
	Illusions [yes, no]
	Human factors [yes, no]
Weather	Time of day [day, night]
	Icing conditions [yes, no]
	Flight rules [IFR, VFR]
Others	Type of flight [transport, recreation, sightseeing flight, training, others, aviation event, testflight]
	Regulations violated [yes, no]
Result	Dead [yes, no]
	Dead [%]
	Dead [Quantity]
	Alive [Quantity] [yes, no]
	Unharmed [yes, no] [Quantity]
	Lightly injured [yes, no] [Quantity]
	Severly injured [yes, no] [Quantity]
	Injury severity points [1,2,3,4]
	Injury severity summarised [light- / severe injuries]

Using significant parameters (p < 0.05) logistic regression (backward stepwise method) was used to calculate the probability of a fatal outcome. Results were implemented in the formula of logistic regression creating a scoring system to calculate the probability of a deadly outcome regarding small aircrafts. All statistical analyses were performed using SPSS® version 27 (IBM®, Armonk, NY, USA).

## Results

**Fig. 1:**
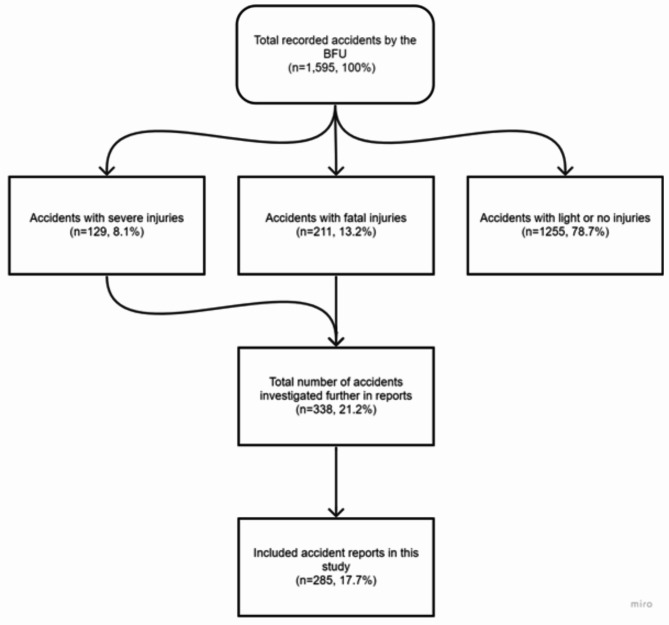
Number of flight accidents that occurred and were investigated. For the present study, 285 severe or fatal accidents were included (n = 129 plus n = 211).

In total, the BFU recorded 1,595 accidents involving small aircrafts under 5,700 kg MTOW in the years 2000 to 2021. From these, 129 (8.1%) cases involved accidents with severe injuries (257 persons affected). 211 (13.2%) accidents could be documented with 390 fatal injuries. Additionally, 1255 accidents with light or no injuries were identified (Fig. 1).

From all recorded accidents involving small aircrafts a total of 338 accidents and serious incidents were investigated in more detail in reports. Based on the given inclusion and exclusion criteria, 285 out of 338 reports were included in this study (Fig. 1).

### Parameters

All parameters collected are listed in Table 1. A taskforce determined parameters as relevant for dispatching in emergency control centres and thus important for efficient care at the scene of an accident. These were selected as core parameters since they can be queried and assessed in the event of an emergency call (Table 1, highlighted). These parameters were further analysed using Chi^2^ -Tests and Mann–Whitney-U-Test to test for correlation with a fatal outcome.

### Outcome parameters

Significant parameters are listed in Table 2. Not included in the analysis were parameters with p-values above 0.05, accordingly months (p = 0.054), days of the week (p = 0.447), number of persons on board (p = 0.112), take-off (p = 0.811), approach (p = 0.051), night-time (p = 0.709) and icing conditions (p = 0.308). Parameters which showed a significant impact (p < 0.05) on a fatal outcome are presented below.

**Table 2:** Quantity of fatal accidents regarding the core parameters.

Category	Subcategory	p-value	Total (n)
Month		0.054	285
	January	0.291	14
	February	0.711	9
	March	0.71	20
	April	0.661	32
	May	0.152	29
	June	0.722	26
	July	0.256	28
	August	0.364	44
	September	0.329	38
	October	0.112	16
	November	0.402	15
	December	0.005	14
Quarter of the year		0.03	285
	First quarter of the year	0.081	43
	Second quarter of the year	0.309	87
	Third quarter of the year	0.04	110
	Last quarter of the year	0.02	45
Weekday		0.447	285
	Monday	0.118	32
	Tuesday	0.403	35
	Wednesday	0.795	42
	Thursday	0.517	29
	Friday	0.443	38
	Saturday	0.930	56
	Sunday	0.112	53
Fire		< 0.0001	285
	No		214
	Yes		71
Over three persons on board		0.112	284
	No		232
	Yes		52
Distance > 10 km away from runway		< 0.0001	284
	No		230
	Yes		54
Flight phase		< 0.0001	284
	Landing	< 0.0001	79
	Cruise	< 0.0001	70
	Take-off	0.811	83
	Approach	0.051	52
Night		0.709	285
	No		261
	Yes		24
Icing conditions		0.308	285
	No		267
	Yes		18

### Last quarter of the year

**Fig. 2:**
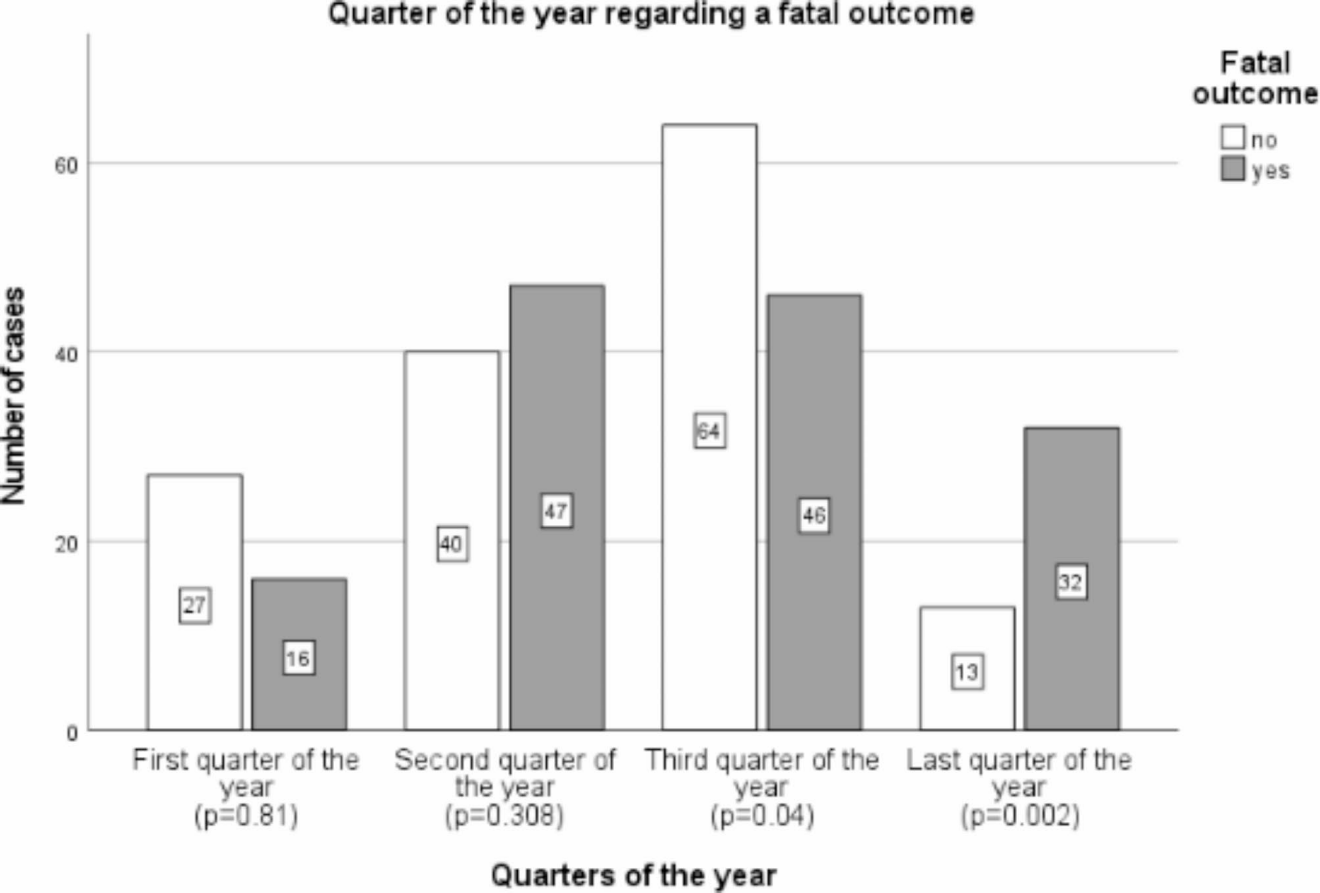
Number of cases grouped by quarter of the year and fatal outcome.

A subdivision in first, second, third, and last quarter of the year (October – December) was performed. Each quarter was tested for correlation with a fatal outcome which showed a significant result for the third (p = 0.04) and last quarter of the year (p = 0.02; Fig. 2).

### Fire

A fire occurred in 71 out of 285 (24.9%) analysed cases of which 60 cases (84.5%) had a fatal outcome. Concerning fire, both in-flight fire and after the accidents is included. For accidents without fire only 81 cases (37.9%) accounted for a fatal outcome. Occurrence of fire was significantly associated with a deadly outcome (p < 0.0001).

### Distance more than 10km away from the runway

The distance from the runway could be evaluated in 284 out of 285 (99.6%) reports. 54 out of 284 (19.0%) were more than 10 km away from the runway of which 43/54 (79.6%) ended fatally. If the distance was below 10 km, the accident was not survived in 98/230 (42.6%). The correlation between an accident occurring at a distance > 10km from the runway and a fatal outcome was tested using Chi^2^ Test and resulted in a p < 0.0001.

### Flight phase

The flight phase could be evaluated in 284 out of 285 reports. In total, 79 accidents (27.8%) occurred during landing which was survived in 64/79 (81.0%) cases. 70 accidents (24.6%) took place during cruise which resulted fatally in 53/70 (75.7%) cases. Accidents during take-off happened in 83/284 (29.2%) events of which 40/83 (48.2%) had a fatal outcome for at least one occupant.

**Fig. 3:**
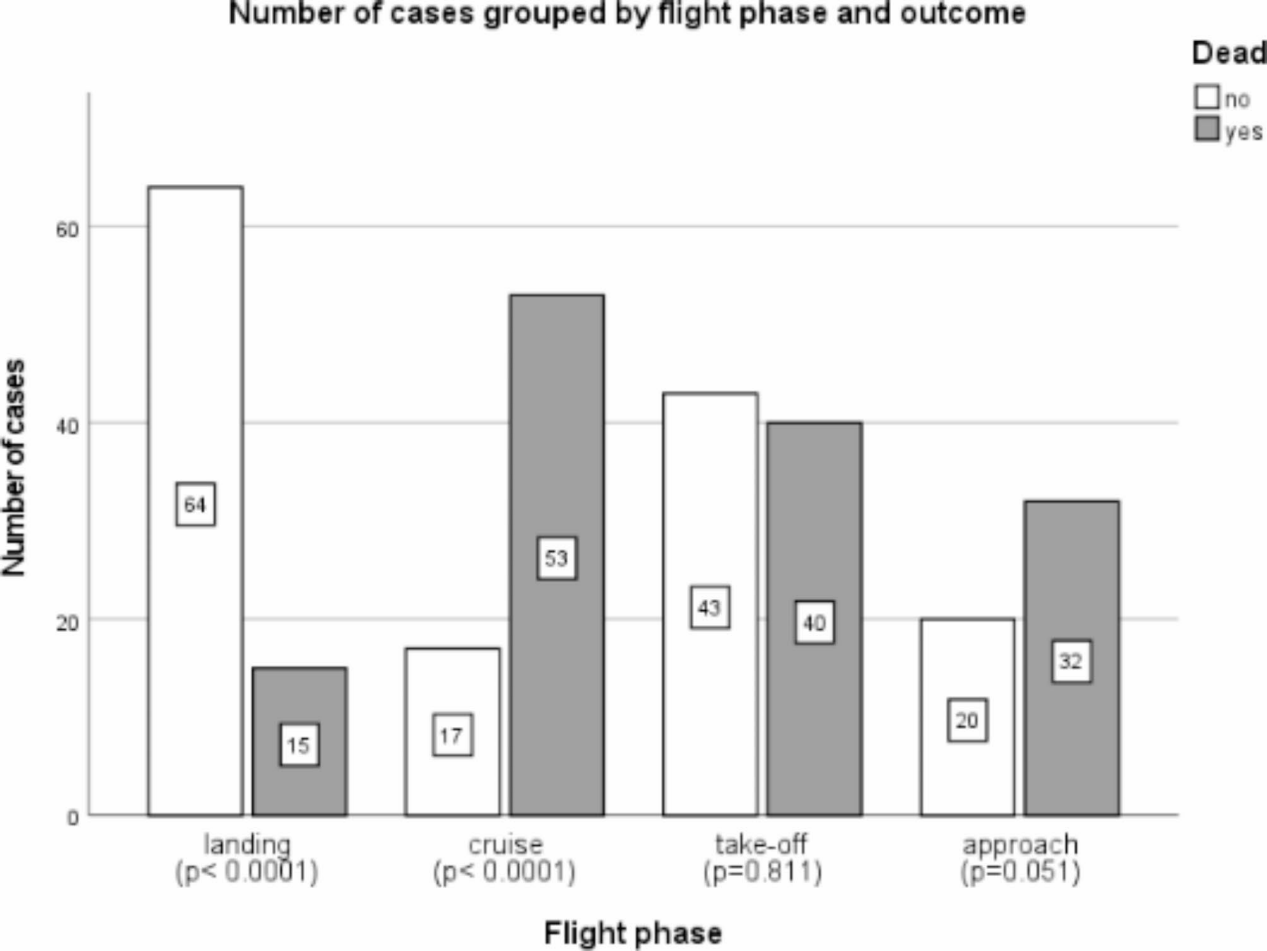
Number of cases grouped by flight phase and outcome, with indication of the p-value.

The least accidents occurred during approach 53/284 (18.7%) for which a fatal outcome was noted in 32/53 (60.4%) instances. The correlation between an accident occurring during the individual flight phases and a fatal outcome was tested using Chi^2^ Test (Fig. 3).

### Score

A model of logistic regression was used to calculate the probability of a fatal outcome. 283 out of 285 (99.3%) cases could be included in the calculation. Using the backward stepwise (B-STEP) Method, four factors remained which are required to calculate the probability of a deadly outcome after an accident with small airplanes.

Significance is shown by Chi^2^ Test with a result p < 0.001. To rate the pattern quality the value of Nagelkerke R^2^ was used. To evaluate the significance of an outcome R^2^ was converted to effect size. With a R^2^ of 0.415 and f^2^ = 0.709 a strong effect is shown (f^2^ > 0.35).

### Description of the parameters and their effect

**Fig. 4:**
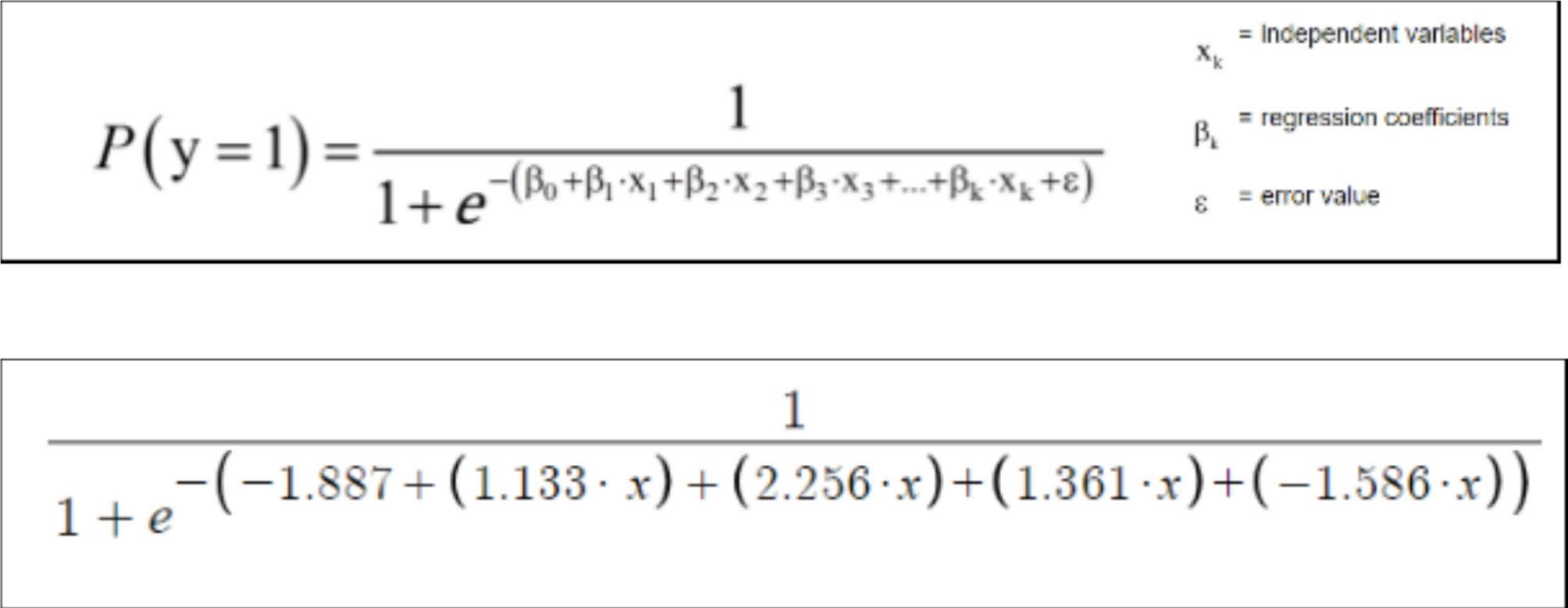
(**a**) and (**b**) Risk calculation formula.

The influence of the variables, in this case on a fatal outcome, is interpreted via the odds ratios (Exp(B)) shown in Table 3^10^. To accurately calculate the probability of a fatal airplane crash using multiple variables the four parameters are inserted into the formula of logistic regression as follows with ε = -1.887 (error term), β_1_ = 1.133 (last quarter of the year), β_2_ = 2.256 (fire), β_3_ = 1.361 (distance > 10 km) and β_4_ = -1.586 (landing) (Fig. 4a, b) ^10^.

**Table 3:** Effect of individual parameters of the score on a fatal outcome.

Parameter	Odds ratio (Exp(B))	95% confidence interval		Effect on a fatal outcome	p-value
		Lower	Upper		
Last quarter of the year	3.106	1.286	7.504	Increased by 210.6%	0.02
Fire	9.544	4.458	20.433	Increased by 854.4%	< 0.0001
Distance greater than 10 km	3.900	1.769	8.599	Increased by 290%	< 0.0001
Landing phase	0.205	0.098	0.427	Decreased by 79.5%	< 0.0001

In case of an accident and applicable parameter, the regression coefficient is multiplied by 1, in case of a non-applicable parameter by 0 (see supplemental data for example).

## Discussion

The present study is the first to evaluate a score to calculate the probability of a fatal outcome regarding fixed-wing motorized aircrafts of less than 5,700 kg MTOW and shows the different effect strengths of individual parameters.

Regarding aviation medicine, Li et al. published an article in the year 2008 analyzing US General aviation accidents to calculate a simple score. The authors were able to identify three main risk factors leading to a fatal outcome after an General Aviation aircraft crash^8^.

Based on these three factors, the authors reported that Fire (F), instrument flight rules/weather (I) and the distance away from an airport (A) are major contributing factors for General Aviation accidents. Using the three parameters, a simple score (each, one point per parameter) can be calculated after the accident.

### Season and the last quarter of the year

An increase in accidents can be seen during the summer months. Small aircrafts are mostly flown for recreational purposes by private pilots, thus more flights occur during the summer months, leading to more accidents during this time. However, this is not accompanied by an increased number of fatal accidents. The proportionally higher number of fatalities in October, November, and December may be attributed to changes in weather conditions coming along with poorer visibility^11^.

According to Li and Baker poor weather conditions provide a ninefold increased risk of a fatal crash. Unlike large aircrafts, small aircrafts are mostly flown under visual flight rules (VFR), so pilots are not proficient when flying in instrument conditions (IFR)^11,12^. A reason for the low fatal outcomes in January, February, and March is not apparent.

Low share of fatal accidents during the first quarter of the year might be explained for example by that only experienced and well-prepared pilots are interested in operating during the coldest season, and the lightest planes stay on ground. However, the number of General Aviation flights as well as data available concerning flying hours per pilot and year is not recorded in any available data base.

### Flight phase

Most accidents with small aircrafts occurred during take-off with 82/284 (29.2%). Accidents during landing occurred in 79/284 (27.8%) cases. This is in accordance with the EASA annual safety review of 2020 in which the two phases take-off and landing recorded the most accidents. However, during landing significantly more accidents (183/448, 40.8%) were reported by the EASA in 2019 than during take-off (76/448, 17.0%)^13^. This confirms that take-off and landing are critical phases for small aircrafts.

The high survival rate of 81.0% in the landing phase in this study can be explained by the fact that accidents in which an emergency landing was still possible were included in this phase. Thus, the landing phase includes all severe incidents in which a landing was possible, as well as accidents in the landing phase.

Unlike during an accident occurring in cruise at high altitude, an accident during landing phase comes along with fewer impact forces that can result in death through direct mechanical effects on the body or fire development. Hill reports deceleration as the most common cause of death when an aircraft hits the ground or water^14^. This also explains the high number of fatal crashes during cruise.

### Fire

The fatal impact of fire after a crash is collectively reported throughout the literature^12^. Within the context of this study, 84.5% of crashes involving fire ended fatally. Li and Baker found a mortality rate of 15% without and 69% with the effect of fire^11^. Within the study carried out in this paper, fire was found in 24.9% of the included cases, but it was associated in 42.6% of fatal cases. This is also consistent with Li and Baker who found general fire development in 13% of small aircraft accidents, which were responsible for 40% of fatalities. Cullen’s results are also consistent with this study, who found fire development in 26% of small aircraft accidents ^14^.

### Distance over 10km from the runway

Studies have already shown an correlation between an accident location away from the airfield and an increased risk of a fatal outcomes^9^,^11^,^15,16^. This is consistent with the results of our study with 79.6% fatal outcomes away from the airfield and a highly significant correlation test. Off-airfield accidents are associated with increased speed and uncontrolled severe impact of the crash on the aircraft. In addition, rescue conditions are usually more difficult^12^.

### Weather

According to Li and Baker^8,11,12^, poor weather conditions increase the risk of a fatal crash. However, in the present study only a quite limited set of weather-related explanatory variables are included in the analysis.

Day/night, icing conditions and IFR/VFR are or course relevant, but also rain, actual visibility, cloud height, wind speed & direction, temperature etc. factors are potentially relevant. However, those parameters are often lacking in the reports and were not sufficiently available for analysis in the present study. In addition to the effect on flying itself, bad weather could, for example, be thought to slow down rescue operations in case of accident.

### Score

To date, our scoring system is the only tool for the rescue coordination centre to objectively assess a GA aircraft crash. The quality of the model could be confirmed by a highly significant Chi^2^ test (p-value < 0.001) and a strong influence strength of f^2^ = 0.709. As shown in supplemental data, the probability of a fatal airplane crash increases substantially when factors are present that have a significant impact on a fatal outcome, whereas a GA crash can be survived to a high probability when none of these factors are present at the time of the crash.

Due to the rapid development in the field of artificial intelligence, scoring systems and objective measurement tools are increasingly in demand to aid in decision making. Projects such as SPELL (semantic platform for intelligent decision and operations support in control and situation centres) are working on the initiation of rapid help in crisis situations with the assistance of artificial intelligence. By providing objective assessment and early detection of critical patients, this score serves as a support and decision-making tool in order to provide the best possible emergency aid in the event of a small aircraft crash^17^.

### General data

Of all 1,595 accidents listed by the BFU, a total of 338 (21.1%) accidents and serious incidents were investigated in more detail in reports. 285 (17.9%) accidents were included in this study. While only every fifth mishap was further investigated in GA accidents, such analyses are routinely undertaken for all commercial aviation Section^18^. There is an absolute need for routine investigations of accidents regarding GA. Knowledge gained from detailed accident reports can be used to effectively improve aviation safety in the future.

### Limitations

The current study is based on retrospective data extracted from the BFU. A possible false transmission of the data was minimized by multiple visual inspections and plausibility checks of the data. The low case number of 285 events can be explained by the selected time-period and the national limitation. Additionally, all accidents not further investigated by the BFU are not represented in this study, leading to a possible bias of the given results.

The time-period investigated was not enlarged, as this would have led to the inclusion of accident cases that are no longer current and thus leading to a distortion of the results. Moreover, there is no other office that produces detailed investigation reports on aircraft accidents in Germany. It can be assumed that the factors identified can be transferred to accidents involving small aircraft in other countries. However, a European and international comparison is required in further studies.

## Conclusion

In the present study, we were able to identify relevant factors associated with a fatal outcome in GA accidents. Even though landing and take-off accounted for most mishaps, landing was shown to decrease the number of fatal outcomes.

The increase in accidents in the last quarter of the year seems to underlie the impact of influencing factors such as poor weather conditions leading to a decreased visibility or deficient runways. The fatal impact of fire and accidents occurring abroad the airport are shown in this study.

As accident analysis is based solely on observable factors there is an absolute need for further investigations. The identified factors could—for the first time—be integrated into a new scoring system and may improve emergency response.

## Declarations

### Availability of data and material

The datasets used and/or analysed during the current study available from the corresponding author on reasonable request.
